# Thermodynamics and Kinetics of Glycolytic Reactions. Part II: Influence of Cytosolic Conditions on Thermodynamic State Variables and Kinetic Parameters

**DOI:** 10.3390/ijms21217921

**Published:** 2020-10-25

**Authors:** Kristina Vogel, Thorsten Greinert, Monique Reichard, Christoph Held, Hauke Harms, Thomas Maskow

**Affiliations:** 1 UFZ - Helmholtz Centre for Environmental Research, Department Environmental Microbiology, Leipzig, Permoserstr. 15, D-04318 Leipzig, Germany; Kristina.vogel@ufz.de (K.V.); monique.reichard@ufz.de (M.R.); Hauke.harms@ufz.de (H.H.); 2 Institute for Drug Development, Leipzig University Medical School, Leipzig University, Bruederstr. 34, 04103 Leipzig, Germany; 3 Laboratory of Thermodynamics, Department of Biochemical and Chemical Engineering, Technische Universitaet Dortmund, Emil-Figge-Str. 70, 44227 Dortmund, Germany; thorsten.greinert@tu-dortmund.de

**Keywords:** biothermodynamics, enzyme kinetics, glycolysis, isothermal titration calorimetry, macromolecular crowding

## Abstract

For systems biology, it is important to describe the kinetic and thermodynamic properties of enzyme-catalyzed reactions and reaction cascades quantitatively under conditions prevailing in the cytoplasm. While in part I kinetic models based on irreversible thermodynamics were tested, here in part II, the influence of the presumably most important cytosolic factors was investigated using two glycolytic reactions (i.e., the phosphoglucose isomerase reaction (PGI) with a uni-uni-mechanism and the enolase reaction with an uni-bi-mechanism) as examples. Crowding by macromolecules was simulated using polyethylene glycol (PEG) and bovine serum albumin (BSA). The reactions were monitored calorimetrically and the equilibrium concentrations were evaluated using the equation of state ePC-SAFT. The pH and the crowding agents had the greatest influence on the reaction enthalpy change. Two kinetic models based on irreversible thermodynamics (i.e., single parameter flux-force and two-parameter Noor model) were applied to investigate the influence of cytosolic conditions. The flux-force model describes the influence of cytosolic conditions on reaction kinetics best. Concentrations of magnesium ions and crowding agents had the greatest influence, while temperature and pH-value had a medium influence on the kinetic parameters. With this contribution, we show that the interplay of thermodynamic modeling and calorimetric process monitoring allows a fast and reliable quantification of the influence of cytosolic conditions on kinetic and thermodynamic parameters.

## 1. Introduction

Reliable and predictable microbial biocatalysts are a prerequisite for the transition from a petro-based to a green bio-based economy. Systems biology uses computational and mathematical approaches to reliably predict and describe the metabolic performances of cells. In addition to omics data, this requires kinetic approaches and considering thermodynamic boundary conditions. Therefore, it is necessary to conduct thermodynamic and kinetic studies of single and combined reactions. One of the most important metabolic pathways is glycolysis. Kinetic investigations, especially for glycolytic reactions, can be found in the literature in large numbers. Some of these reactions have been monitored using calorimetry [1,2,3,4,5,6]. Isothermal titration calorimetry (ITC) is well suited for such measurements. In this method, a heat flow generated during the enzymatic reaction is monitored [3,7,8,9,10,11,12,13] and the heat trace is used to quantify the reaction enthalpy change and kinetic parameters. Calorimetry is non-invasive, and neither chemical modification nor stabilization of the reactants and enzymes are necessary [9,10,14]. Thus, calorimetry allows the fast screening for the influence of different cytosolic conditions on metabolic reactions. Especially in the past, the influence of cytosolic conditions on reactions was mostly neglected in the literature. A general overview about the fundamental influence of the solvent on organic reactions can be found in the following literature [15,16,17].

Some newer publications have shown that metabolic network considerations depend strongly on the conditions in the cytosol [3,18,19,20,21]. Important cytosolic conditions are pH, ionic strength, temperature, Mg^2+^ and crowding by macromolecular agents. The cytosolic pH, for example, depends on the considered organism. For instance in human cells the pH varies between 7.3 and 7.4, whereas in prokaryotic cells intracellular pH values between 4.5 and 9.5 are reported [22]. In 2010, Tadashi Ando published a paper stating that the total salt concentration in cells is 150 mM [23], others reported a value of 300 mM [24]. The concentration of magnesium ions (bound and free) varies between 0.1 and 30 mM, depending on the organism investigated [25,26,27]. The influence of macromolecular crowding is worth mentioning in particular, as cells contain between 200 and 300 g L^−1^ of proteins [28]. Therefore, it is not surprising that this influence can significantly change both the thermodynamic state variables and kinetic parameters of the reactions [3,6,29] compared to pure buffer solutions. Another important factor that has often been neglected is the reversibility of some glycolytic reactions. In these reactions, equilibrium between reactants and products is obtained. This means that not all reactants are converted into products. This fact must be considered particularly for kinetic data evaluation. In part I, we were able to show that two different models from irreversible thermodynamics described the reaction progress and the kinetic approximation to the thermodynamic equilibrium very well and that the kinetic constants fulfill the Arrhenius relation below deactivation [30]. Both models need—similar to the conventional Michaelis-Menten (M-M) model—the reaction rate as a function of the substrate concentration. The first model developed by Noor et al. combines the conventional M-M model with an approach from irreversible thermodynamics [31]. This model, however, needs three adjustable parameters in the original development. It can be simplified assuming single enzyme saturation resulting in two adjustable parameters. The simplification was proven to describe the example reactions very well. The second (flux-force) model relates the thermodynamic driving force with the reaction kinetics [31,32] using a single fit parameter. A single parameter allows a simple assignment to changes in cytosolic conditions. ITC measurements allow the fast and easy monitoring of the reaction progress for the different conditions.

Therefore, ITC reaction monitoring and models from irreversible thermodynamics (Noor and flux-force) have been combined in this work. As reversible model reactions, the reactions 2 (uni-uni mechanism) and 9 (uni-bi mechanism) of glycolysis were investigated. Reaction 2 is the isomerization of glucose 6-phosphate (G6P) to fructose 6-phosphate (F6P) by the enzyme phosphoglucose isomerase, Equation (1). Reaction 9 is the conversion of 2-phosphoglycerate (2PG) to phoshoenolpyruvate (PEP) and water by enolase, Equation (2).

(1) G6P  ←→ F6P 

(2) 2PG  ←→ PEP+ H2O 

Single injection measurements were performed and both the enthalpy change of the reaction and the kinetic parameters were determined [8,12,31,32,33,34]. To investigate the influence of cytosolic conditions, measurements were performed at different temperatures, pH values, concentrations of different salts, and with macromolecular crowding agents.

## 2. Results

### 2.1. Influence of the Cytosolic Conditions on the Reaction Enthalpy Change

**Figure 1:**
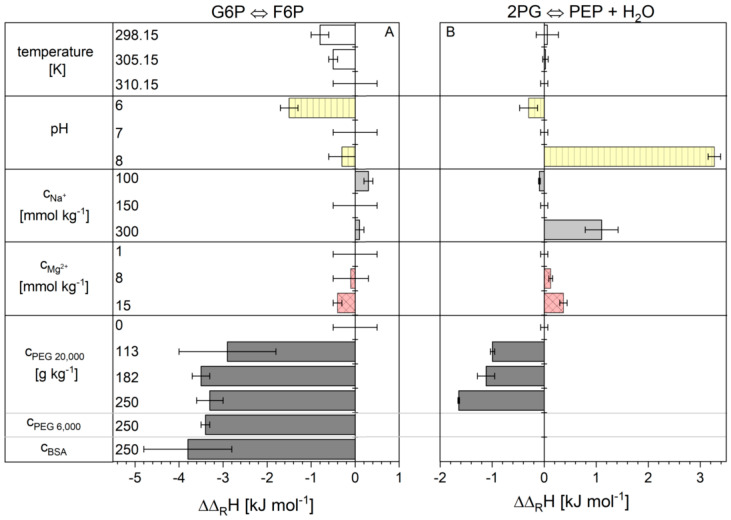
Difference ($\Delta \Delta_{R}H$) between the reaction enthalpy changes under cytosolic conditions and basic conditions reaction 2 (**A**) and 9 (**B**). The reaction enthalpy change under basic conditions 11.1 ± 0.5 kJ mol^−1^ for reaction 2 and 2.4 ± 0.1 kJ mol^−1^ for reaction 9. The error bars result from the standard deviation of the triple determination of each measurement condition.

ITC is a special calorimetric method that combines the addition of substrates or enzymes with minimal thermal disturbances with the real-time tracking of the reaction progress in µW ranges. It offers the advantage of providing both the thermodynamic state variable reaction enthalpy change and kinetic parameters. In this work, the reactions of F6P to G6P and PEP to 2PG (for practical reasons the opposite direction of the glycolysis) were measured and evaluated. For both reactions, the reaction enthalpy change was determined with Equation (3) (Section 4). The values for the total reaction heat Q were measured using ITC and the equilibrium ratio $K_{c}$ values were obtained using ePC-SAFT. The reaction enthalpy changes for reactions 2 and 9 are given in Tables S3 and S4 in the supplementary materials. For reasons of clarity, Figure 1 shows the deviations from the reaction enthalpy change under conditions, named basic conditions, where the reaction enthalpy changes of 11.1 ± 0.5 kJ mol^−1^ for reaction 2 and 2.4 ± 0.1 kJ mol^−1^ for reaction 9 were obtained. “Basic conditions” are considered as reference states and are defined in detail in the Section 4.

The temperature and the concentrations of sodium and magnesium ions did not have a significant influence on the reaction enthalpy change of reaction 2 in the cytosolic range. The situation is different with the pH value. Reaction enthalpy changes obtained at pH 7 and 8 cannot be distinguished statistically, whereas at pH 6 a lower reaction enthalpy change of 9.6 ± 0.2 kJ mol^−1^ was observed. The addition of PEG 20,000 as a crowding agent had the greatest influence on the reaction enthalpy change. Already, an addition of 113 g kg^−1^ lowered the value to 8.2 ± 1.1 kJ mol^−1^. The increase of the PEG concentration to 250 g kg^−1^ for PEG 20.000 or PEG 6000 and a BSA concentration of 250 g kg^−1^ did not change the reaction enthalpy change significantly compared to 113 g kg^−1^. The influence of the cytosolic conditions on the reaction enthalpy change of reaction 9 was different for some conditions (Table S4 in supplementary materials) [6]. A reaction enthalpy change of 2.4 ± 0.1 kJ mol^−1^ was determined under basic conditions. A change of temperature had no influence on the reaction enthalpy change. With increasing pH and higher concentrations of sodium and magnesium ions, the reaction enthalpy change increases. Crowding again had a strong effect. With increasing PEG 20,000 concentrations, the value for the reaction enthalpy change dropped to 0.7 ± 0.02 kJ mol^−1^.

### 2.2. Influence of the Cytosolic Conditions on the Reaction Kinetics

#### 2.2.1. The Noor Model

**Figure 2:**
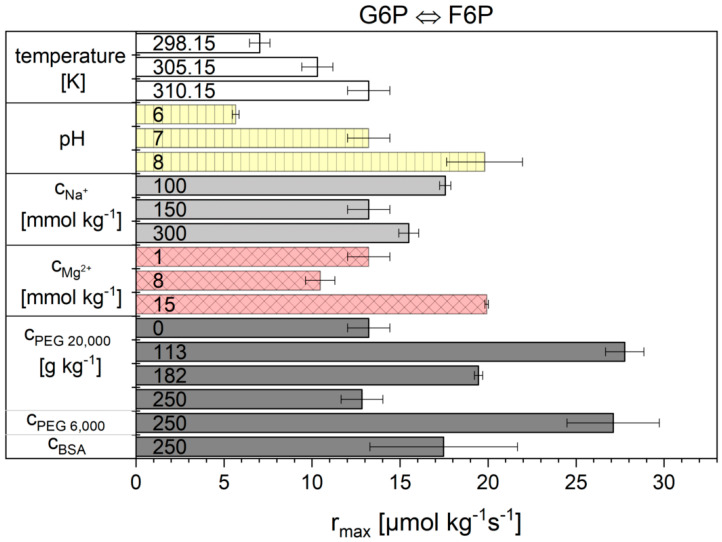
$r_{max}$ values obtained for reaction 2 from the Noor model. The error bar results from the standard deviation of the triple determination of each measurement condition.

**Figure 3:**
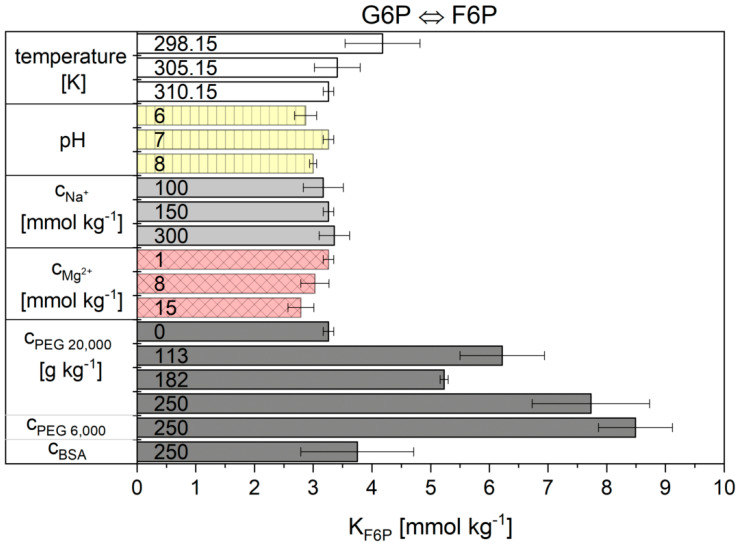
$K_{F6P}$ values obtained for reaction 2 from the Noor model. The error bar results from the standard deviation of the triple determination of each measurement condition.

Noor’s model is based on reversible Michaelis-Menten kinetics and subdivides the kinetic model into three factors: the maximal rate, the enzyme saturation level and the thermodynamic driving-force [31]. This model was already successfully tested and the temperature dependencies were described in previous work [30]. In this work, the influence of cytosolic conditions on the kinetics of reactions 2 and 9 was investigated. The results for the parameters $r_{max}$ and $K_{F6P}$ from Equation (11) for reaction 2 are shown in Figure 2 and Figure 3 and are listed numerically in Table S5 of the supplementary materials. The kinetic parameter $r_{max}$ under basic conditions was 13.2 ± 1.20 µs^−1^. Unfortunately, unambiguous dependencies of the kinetic parameter on the other conditions investigated (sodium and magnesium ion concentration and crowding) were not obtained. Both molecular weight and concentration of the applied crowding agents caused non-linear effects on the parameter $r_{max}$.

The situation was different for the fit parameter $K_{F6P}$. This value obviously remained constant for almost all conditions examined except for solutions containing crowding agents. With increasing concentrations of the crowding agent, the $K_{F6P}$ more than doubled for the highest concentrations of PEG. The $K_{F6P}$ value did not change significantly upon addition of BSA compared to basic-conditions.

**Figure 4:**
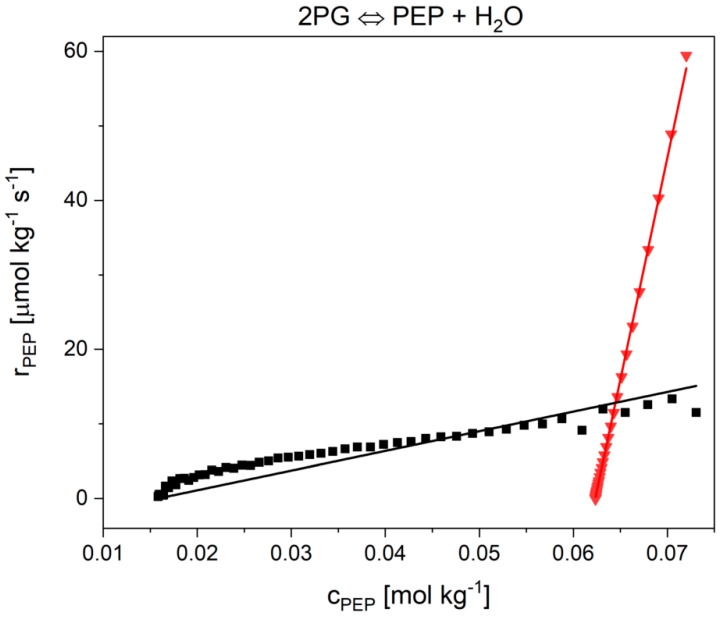
Fitting of the kinetic data (scatter) with the Noor model (solid lines). The adjustable parameters were Λ and $K_{2PG}$. Reaction in the presence of high crowding agent concentrations of 250 g kg^−1^ PEG 20.000 (black squares) and 15 mmol kg^−1^ MgCl_2_ (red triangles). The fitting parameters for 250 g kg^−1^ PEG 20.000 are Λ= 0.11 ± 0.01 ms^−1^ and $K_{2PG}$ = (1.56 ± 2.2) × 10^24^ mmol kg^−1^ and for 15 mmol kg^−1^ MgCl_2_ Λ= 1.10 ± 0.10 ms^−1^ and $K_{2PG}$ = (−4.91 ± 5.69) × 10^16^ mmol kg^−1^.

In part I it was shown for reaction 9 that the Noor model described the reaction progress for basic-conditions accurately [30]. In the model two parameters were required. Unfortunately, we found that the model has weaknesses for two cytosolic conditions (Figure 4), namely for high PEG and high Mg^2+^ concentrations. The value of the $K_{2PG}$ parameter (−4.91 ± 5.69 × 10^16^ mmol kg^−1^) at 15 mmol kg^−1^ magnesium ions (the fastest reaction investigated) was not statistically significant. However, the model described the experimental data very well. In the presence of 15 mmol kg^−1^ MgCl_2_, it is noticeable that the modeled curve was found to be linear. The linear behavior—which was observed only in the presence of high magnesium concentration—was probably responsible for the scattering of the fit parameter $K_{2PG}$. Another weak point of the model became obvious after fitting the model to the measurement with 12.5 mmol kg^−1^ PEG 20,000 (Figure 4 black squares, the slowest investigated reaction). Here, the model (dashed gray line) did not match the experimental data and again the $K_{2PG}$ value ((1.21 ± 1.71) × 10^23^ mol kg^−1^) was not statistically significant. The curvature of the experimental data (convex) was different to the curve (concave) under basic cytosolic conditions (Figure S1 in supplementary materials). 

**Figure 5:**
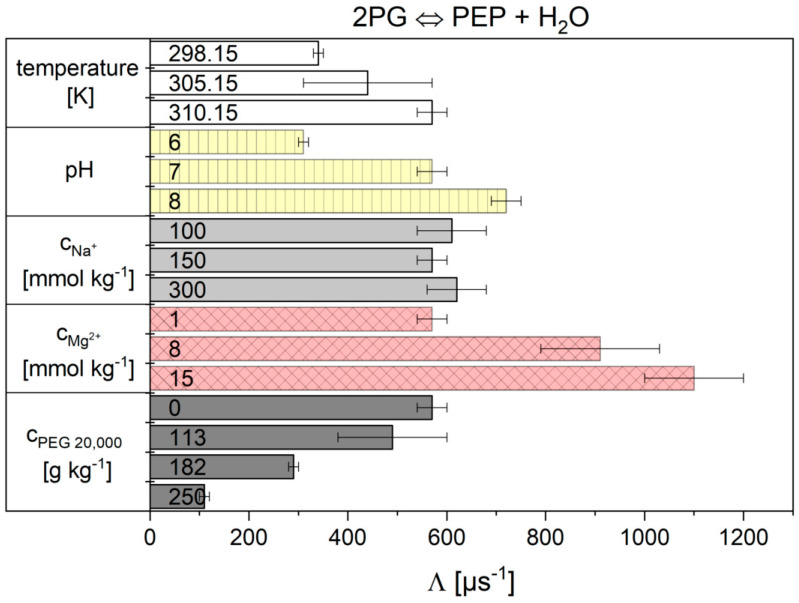
Values obtained for reaction 9 from the Noor model. The error bar results from the standard deviation of the triple determination of each measurement condition.

**Figure 6:**
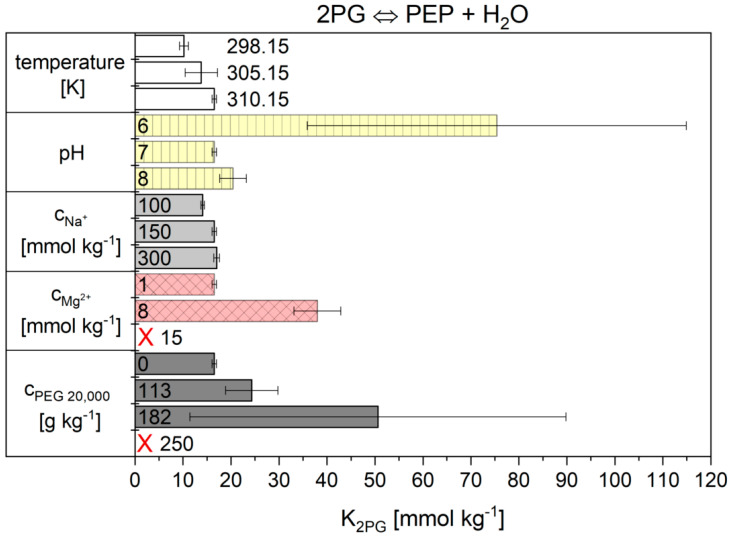
$K_{2PG}$ values obtained for reaction 9 from the Noor model. The error bar results from the standard deviation of the triple determination of each measurement condition. The red crosses mark the two conditions where no significant fit parameters were obtained.

The influence of all tested cytosolic conditions on the parameters $K_{2PG}$ and Λ from Equation (12) of the Noor Model are given in Figure 5 and Figure 6 and numerically in Table S6 in supplementary materials. Dependencies on temperature, pH, magnesium ions and crowding were observed. The concentration of sodium ions had no influence. The $K_{2PG}$ values obtained for reaction 9 from the Noor model have great difficulty in producing significant values under some conditions. At pH 6 and 182 g kg^−1^ PEG 20,000, the error bars are very large. At 15 mmol kg^−1^ Mg^2+^ and 250 g kg^−1^ PEG 20,000 the values are not statistically relevant. But the main problem with the Noor Model was that two parameters can compensate each other and thus make a clear description of the cytosolic influence difficult. Therefore, only the flux-force model is used in the following.

#### 2.2.2. The Flux-Force Model

**Figure 7:**
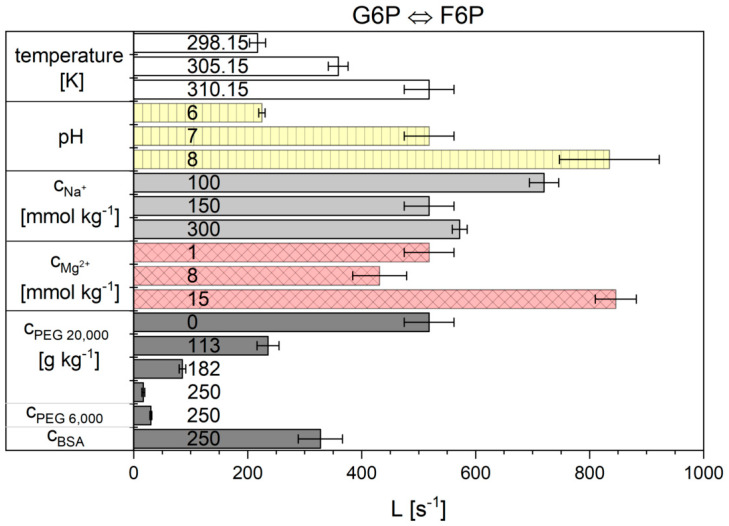
Influence of the different cytosolic conditions on the kinetic parameter *L* of the flux-force model of reaction 2.

The flux-force model for kinetic analysis needs only a single parameter to describe the reaction progress, which can be compared under all measured cytosolic conditions. In part I it was shown that this model describes the reaction progress very well and that *L* fulfills the Arrhenius relation [30]. The origin and the message of the kinetic fit-parameter *L* is described in the Section 4. *L* is obtained by the slope of the linear regression of the plot reaction rate against $\frac{\iota }{2-\iota }$. With $\frac{\iota }{2-\iota }$ values from 0–0.6, a wide linear range is obtained. This evaluation was performed for each measurement. The *L* parameter values obtained for reaction 2 are shown in Figure 7, the numerical values can be found in Table S7 in supplementary materials.

It is shown that the kinetic parameter *L* of reaction 2 depends much more on the conditions than the reaction enthalpy change. With increasing temperature (between 298.15 K and 310.15 K) the value for *L* increased almost 2.4-fold. The pH raised *L* by a factor of approximately 4 and Mg^2+^ increased it 2-fold. The concentrations of sodium ions, however, did not show such a clear dependence. A decrease in the concentration from 150 to 100 mmol kg^−1^ increased the *L* parameter, whereas an increase to 300 mmol kg^−1^ did not significantly change *L* compared to the basic-conditions. The addition of each of the crowding agents significantly decreased *L*. A higher molar mass of PEG led to a stronger reduction in the reaction rate and of *L*. The influence of the linear PEG molecule was indeed found to be stronger than that of the more spherical protein BSA.

**Figure 8:**
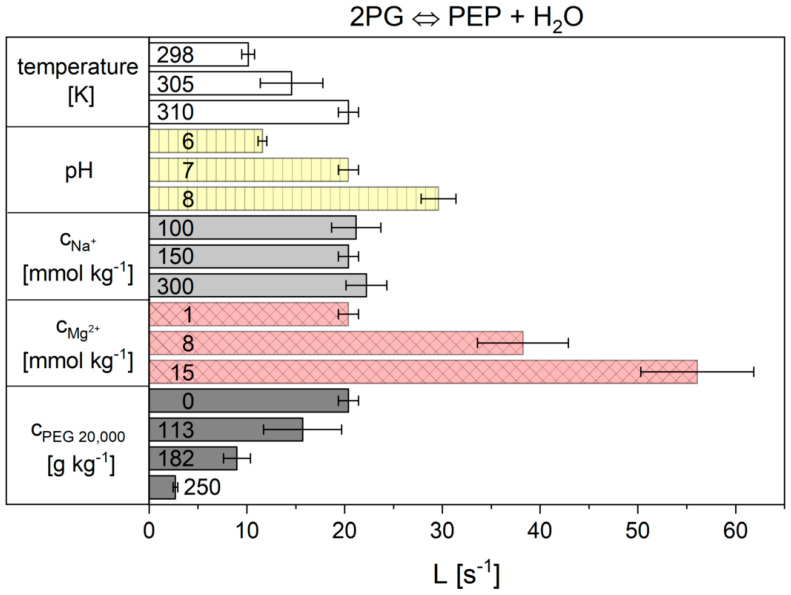
Influence of the different cytosolic conditions on the kinetic parameter of reaction 9 of the flux-force model.

The overall results for all individual cytosolic conditions (temperature, pH, concentrations of Na^+^, Mg^2+^ and crowding agent) of reaction 9 are summarized in Figure 8. *L* increased with the rising temperature. Similarly, the pH value influenced the reaction kinetics, since a pH increase to pH 8 led to an increase in the *L* parameter. The concentration of sodium ions had no statistically significant influence on *L*. In contrast, the concentration of magnesium ions strongly influenced *L*. By increasing the Mg^2+^ concentration, the value of *L* increased considerably, which corresponds to a dramatic increase in the reaction rate. The exact opposite was observed when crowding agents were added. Addition of PEG 20,000 as a crowding agent led to a dramatic reduction of *L* and thus to a strong decrease in the reaction rate. The exact values and errors resulting from the triple determination are given in supplementary materials (Table S8).

## 3. Discussion

### 3.1. Influence of the Cytosolic Conditions on the Reaction Enthalpy Change

The reversible reactions 2 and 9 of glycolysis were monitored using ITC, because ITC provides both the reaction enthalpy change and kinetics in a simple manner. First, we discuss reaction 2. Under basic conditions, $\Delta_{R}H$ was 11.1 ± 0.5 kJ mol^−1^. Comparable values of 12.05 ± 0.2 kJ mol^−1^ [1], 11.7 ± 0.2 kJ mol^−1^ [4] and 10.8 kJ mol^−1^ [35] were reported in the literature. When measuring at different pH values, it was observed that the reaction enthalpy change reduced to 9.6 ± 0.2 kJ mol^−1^ at pH 6. The reason for this could be the pH-dependent speciation of reactants. It is assumed that G6P^2-^ and F6P^2-^ are the reacting species [1]. Between pH 6 and pH 7, their proportion of G6P^2-^ decreases from 79% to 28% and the proportion of F6P^2-^ decreases from 84% to 35%. The increase in the pH value to 8 did not bring about any significant change. Taking the differently protonated species and their formation enthalpy changes [36,37] the pH dependency of the reactions can be predicted. The same trend of the pH dependence was obtained when calculating the reaction enthalpy change (Table 1). To compare the values at different pHs, the reaction enthalpy change at pH 7 was set to 100%. A reduction of the reaction enthalpy change to 91% at pH 6 was calculated, using ITC at a value of 86% was determined. The pH increase to 8 increased the reaction enthalpy change to 101% (prediction) or to 97% (ITC).

The concentrations of sodium and magnesium ions did not influence the reaction enthalpy change. The addition of crowding agents on the other hand led to a drastic reduction of the reaction enthalpy change. PEG 20,000, PEG 6000 and BSA were used as models for crowding agents. At the highest concentration of crowding agents (250 g kg^−1^) the reaction enthalpy change decreased to 70 ± 3% (PEG 20,000), to 69 ± 1% (PEG 6000) and to 66 ± 9% (BSA). The influence of cytosolic conditions on the reaction enthalpy change of reaction 9 has already been investigated in a previous paper where we also showed that cytosolic conditions have a strong effect on the reaction enthalpy change, especially pH, high sodium ion concentration and crowding [6]. When comparing the results of both reactions, it is noticeable that the change in pH value and the introduction of crowding agents had the greatest influence on the reaction enthalpy change. In both reactions we observed a reduction in the reaction enthalpy change when the solvent was crowded by macromolecules. Similar results were reported for a trypsin-catalyzed reaction using PEG 600 and PEG 6000 [18] and for the glycolytic hexokinase reaction using 250 g L^−1^ BSA [3]. The authors found the effect in four different reactions and with different crowding agents. Unfortunately the reasons for this result were not discussed in the respective publications. Three explanations for the influence of PEG are imaginable [6]. The Gibbs free energy change of the reaction ($\Delta_{R}G$) is composed of the reaction enthalpy change and the entropy change (Equation (7) in the Section 4). The addition of crowding agents has a strong influence on the entropy change, which is called an excluded volume effect. This means that crowding reduces the available reaction volume in the solution. This also reduces the degrees of freedom for molecular movement und thus the reaction entropy change. The calculated values for the three thermodynamic state variables are shown in Table 2.

The values of the Gibbs free energy change increases for both reactions when crowding agents are introduced. At the same time, the values of the reaction enthalpy change decreased. This leads to the fact that for both reactions a significant decrease in entropy change with crowding agents results. Therefore, the reaction is less entropy-driven in the presence of crowding agents than without and this effect is partly cancelled out by weaker obstruction by the reaction enthalpy change [6]. Second, there could be an interaction between the reacting species and the crowding agents. For PEG, repulsive interactions become stronger with increasing molecular mass of PEG [38]. This repulsive interaction supports the hypothesis of the excluded volume. Third, there is a reduction in the dielectric constant of the solution by the addition of crowding agents, which leads to stronger long-range electrostatic forces between the metabolites. This increases the influence of the ionic strength on the activity coefficients and thus also on the reaction enthalpy change. The dielectric constant of water ($\varepsilon_{r}$ = 81 at 20 °C) is higher than the value for PEG or proteins found in the literature. Though no value for PEG 6000 or PEG 20,000 are published, a value of 11.6 at 20 °C has been reported for PEG 400 9 [39]. It has been shown that high concentrations of PEG significantly reduces the dielectric constant of water [39,40]. The dielectric constant of proteins was also investigated [41,42,43]. Large proteins have a low dielectric constant inside the protein ($\varepsilon_{r}$ = 6–7) and of $\varepsilon_{r}$ = 20–30 at the surface [42]. The interaction of these 3 effects could explain the effect of crowding on the reaction enthalpy change.

### 3.2. Influence of the Cytosolic Conditions on the Reaction Kinetics

#### 3.2.1. Validation of Kinetic Models

In this paper, two kinetic models were used for data analysis to verify whether both are able to describe the influence of cytosolic conditions on the kinetics. The Noor model could not describe all conditions. For reaction 2, the Noor model was not able to show a clear dependence of macromolecular crowding. This is because the two parameters of Noor behaved differently under the influence of the cytosolic conditions. For reaction 9, if the Mg^2+^ and crowding agent concentrations are too high, the model did not provide significant kinetic parameters because the error was greater than the calculated value. For all other conditions, both models showed exactly the same trend. The flux-force model proved to be successful for the analysis of our whole data set. Therefore, the following discussion is based only on the values obtained from the flux-force model.

#### 3.2.2. Importance of Cytosolic Conditions

To determine the influence of the individual conditions, only one parameter was changed in each measurement, starting from the basic conditions with 0.2 mol kg^−1^ MOPS buffer, 0.15 mol kg^−1^ Na^+^, 0.001 mol kg^−1^ MgCl_2_ and pH 7 at 310.15 K. The definition is based on the fact that these conditions are most similar to cell conditions, apart from the concentration of crowding agents. Normally the cell contains different salts. For simplification, we have used sodium chloride as model salt because the purchased substrates are also sodium salts. The typical concentration of sodium chloride is about 5 mM [44]. To mimick macromolecular crowding, PEG and BSA were chosen as suggested by the literature [3,45,46]. In the literature [3,45,46,47,48,49], similar effects of the crowding on kinetics are described. For instance, Rohwer observed in 1998 that crowding reduced the dissociation rate constants of enzyme complexes [46]. Olsen examined the kinetics of hexokinase in a concentration range from 0 to 250 g L^−1^ of BSA as the crowding reagent. Olsen et al. found a reduction of K_S_ and k_cat_ with the increasing concentration of the crowding agents [3]. The reduction of K_S_ was explained by the decrease in the ratio of activity coefficients between the enzyme–substrate complex and the native enzyme with increasing BSA concentrations. Three different reasons for the reduction of the reaction rate in the presence of crowding agents are possible. First, crowding has been described to have a size exclusion effect, which means that the reaction volume is reduced by the addition of crowding agents [50]. This should on one side enhance the reaction rate. On the other side, the path length of movement of the reacting molecules is increased, as they have to bypass the crowding agents [20]. The molecule size determines the strength of the effect. This again leads to a slowdown in the molecular diffusion rates [50,51]. Indeed, it was found that increasing PEG concentrations causes a reduction in tracer diffusion [45]. Second, the Stokes–Einstein Equation describes an inverse relationship between the diffusion coefficient and the radius of the macromolecules. Third, electrostatic and van der Waals interactions between the macromolecules and the reacting species may also affect the reaction rates.

#### 3.2.3. Influence on Reaction 2

For reaction 2, with a rising temperature and pH, the parameter *L* rises. A comparable result was published by Dyson et al., who have determined a maximum reaction rate at 313.15 K [35]. From part I we know that the temperature influence in the investigated range can be described by the Arrhenius relation. The pH dependence also fits to literature values, which indicates a pH optimum of 8.5 [1,35,52]. The increase in sodium ion concentration from 150 to 300 mmol kg^−1^ seems to have no significant influence, whereas the decrease to 100 mmol kg^−1^ increases the reaction rates. Therefore, it can only be assumed that the sodium ions have a possible inhibitory effect on the enzyme. The influence of magnesium ions is similarly difficult to interpret. An increase to 8 mmol kg^−1^ seems to lower the value and a further increase to 15 mmol kg^−1^ then increases the value markedly. Macromolecular crowding has the strongest influence of all cytosolic conditions on the kinetics of the reaction. Already, an addition of 113 g kg^−1^ PEG 20,000 reduces the value of *L* to 45 ± 0.04%. An increase to 250 g kg^−1^ leads to a drastic reduction to 3 ± 0.05% of the value at basic conditions. In order to investigate whether the molecular mass of the crowding agents changes the strength of the influence on the kinetics, a crowding agent concentration of 250 g kg^−1^ PEG 6000 was also used. It can be observed that there is also a strong reduction of the *L* value to 58 ± 0.3%, but not as strong as with PEG 20,000. BSA was used as another crowding agent. Compared to PEG, the influence of BSA on the kinetics of the reaction was not so dramatic. There is a reduction of *L* to 63 ± 8%. This effect can be explained by the shape of the molecules. BSA has a 3D structure that resembles a sphere and the molecule is tightly packed in a compact structure. PEG 6000 and 20,000 are in loose coil structures, which are very flexible [53]. This flexibility of the PEG structure leads to such a large reduction of the kinetics. For a more in-depth analysis, other effects such as the charge of the crowing agents, the enzyme under investigation and the metabolite should of course also be taken into account.

#### 3.2.4. Influence on Reaction 9

With an increasing temperature, *L* increases according to the expectations. From part I it is know that the temperature behavior can be described by the Arrhenius model [30]. Our observed temperature dependency is consistent with Westhead’s studies [54]. Another work also reports a similar temperature behavior at elevated temperatures for an octameric thermophilic enolase [55]. In the range between 100 and 300 mmol kg^−1^ Na^+^ no statistically significant influence on the kinetics was observed. In slight contradiction to our results, one publication reported an inhibition of enolase activity by 20% at high salt concentrations (100 mM to 1000 mM) [56]. However, in accordance with this publication, we could see a strong increase in the reaction rate in the absence of sodium chloride (the heat curve is shown in Figures S1 and S2). Unfortunately, the reaction without sodium chloride became too fast to be quantifiable with our instrumentation and model. Magnesium ions in the range between 1 and 15 mmol kg^−1^ have a strong activating effect on the enolase reaction (increase up to 175 ± 0.3% of the basic conditions). Wold and Warburg observed a similar activation of yeast enolase by magnesium ions [57,58]. They reported a 23% enhanced rate when increasing the magnesium ions concentration from 1 mM to 16 mM instead of 175 ± 0.3% [57,59]. In our measurements, an increase of the kinetics from pH 6 to 8 was observed. Our results match Wold’s studies who reported a similar pH effect. The enolase studied by Wold showed a maximum activity at about pH 7.8 [59]. Finally, crowding agents have a particularly strong influence on the *L* parameter for the enolase reaction. Here, *L* is reduced to 13 ± 0.01% in the presence of typical concentrations of crowding agents in the cytosol.

## 4. Materials and Methods 

### 4.1. Chemicals

Yeast enolase, monosodium phosphoenolpyruvate, phosphoglucose isomerase type III from baker’s yeast and bovine serum albumin (BSA) were purchased from Sigma Aldrich (Sigma-Aldrich Chemie GmbH, Steinheim, Germany), fructose 6-phosphate disodium salt from Alfa Aesar (Thermo Fisher (Kandel) GmbH, Kandel, Germany), MOPS from AppliChem (AppliChem GmbH, Darmstadt, Germany), sodium chloride from CHEMSOLUTE (Th. Geyer GmbH & Co. KG, Renningen, Germany), polyethylene glycol with an average molar mass of 20,000 g mol^−1^ (PEG 20,000) from Merck (Merck KGaA, Darmstadt, Germany), polyethylene glycol 6000 from Serva (SERVA Electrophoresis GmbH, Heidelberg, Germany), magnesium chloride hexahydrate and sodium hydroxide from Roth (Bernd Kraft, Duisburg, Germany). An overview of all used chemicals, the CAS-numbers and purity is given in Table S1 in the supplementary materials.

### 4.2. Sample Preparation

For calorimetric measurements, two solutions had to be prepared; one solution containing the enzyme and the other the substrate. Sample preparation was done according to [30]. The influences of temperature, pH, concentrations of sodium and magnesium ions, and the influence of crowding agents were investigated (Table 3). The reaction buffer was varied according to the conditions under investigation. A “basic condition” was defined, in which the buffer contains 0.2 mol kg^−1^ MOPS buffer with 0.15 mol kg^−1^ Na^+^, 0.001 mol kg^−1^ Mg^2+^, pH 7 and 310.15 K. This basic condition were taken as a starting point for the measurements of the different cytosolic influences. Only the change of one condition per measurement was determined with all others remaining as in the basic conditions.

### 4.3. ITC Measurements of the Phosphoglucose Isomerase Reaction

All experiments were conducted using the PEAQ-ITC from Malvern Panalytical (Malvern, UK). Single injection measurements were performed with a 100 mmol kg^−1^ F6P solution in the syringe and a 15 nmol kg^−1^ PGI solution in the reaction cell [30]. Multiple injection measurements were not performed for two reasons: (1) reactions were so fast that no plateau between the single injections could be reached and (2) the enzyme concentration could not be reduced because the instrumental thermal power change would have been too small. The setup of the PEAQ-ITC was set to high feedback, reference power of 40 µW, stirrer speed of 750 rpm and a titration speed of 0.5 µL s^−1^ and a baseline recording of 15 min. Two injections were performed and the heat signal was allowed to return to the baseline, indicating the end of the reaction. The first injection of 0.4 µL was not included in the evaluation. The waiting time until the next injection was 10 min. The second injection titrated 10 µL. Reference measurements of F6P titrated to buffer were performed to consider the influence of the heat of dilution. All measurements were performed as triplicates.

### 4.4. ITC Measurements of the Enolase Reaction

Single injection measurements were also performed here. The syringe was loaded with 12 µmol kg^−1^ enolase and the reaction cell with 89.5 mmol kg^−1^ PEP. The setup of the PEAQ-ITC was set to high feedback, reference power of 41.9 µW, stirrer speed of 750 rpm, titration speed of 0.5 µL s^−1^, baseline recording of 15 min and an injection volume of 39.2 µL. Reference measurements of PEP titrated to buffer were performed to consider the influence of the heat of dilution. All measurements were performed as triplicates.

### 4.5. Thermodynamic Calculations

As described by Vogel et al. [30], the initial phase of the measurement was not taken into account due to the thermal inertia of the ITC and the heat of dilution. The reaction enthalpy change can be calculated from the observed heat production rate P, Equation (3).

(3) $$\Delta_{R}H=\frac{\int_{0}^{\infty }P\left(t\right)\mathrm{dt}}{\left(c_{S}^{0}{-c}_{S}^{\mathrm{eq}}\right)*m\;}$$

with $c_{S}^{0}$ as the substrate concentration (in mol kg^−1^) in the cell after injection and m as the mass of the reaction volume in the calorimetric cell (in kg). $c_{S}^{eq}$ is the substrate concentration in the equilibrium (in mol kg^−1^) and was calculated from the apparent equilibrium constant $K_{c}$. $K_{c}$ was calculated from reactant and product ($c_{P}^{eq}$) concentration, Equation (4).

(4) $$K_{c}=\;\frac{c_{P}^{\mathrm{eq}}}{c_{S}^{\mathrm{eq}}}$$

In this work, $K_{c}$ was not determined experimentally as described by Vogel et al. [30] A direct measurement of the material from the ITC was found to be error-prone due to the very small reaction volume and the high reactant concentration. These samples need to be highly diluted, which leads to large errors in the equilibrium concentrations. $K_{c}$ was calculated from the thermodynamic equilibrium constant $K_{a}^{}$ from previous works [1,6], which were determined in parallel measurements outside of the ITC. The activity-coefficient ratio was $K_{\gamma }$, which was predicted with ePC-SAFT in this work, Equation (5).

(5) $$K_{a}{=\;K}_{c}\cdot {\;K}_{\gamma }$$

While $K_{c}$ and $K_{\gamma }$ depend on the reaction solution (i.e., concentrations of products, reactant, and all other components), $K_{a}$ is a true constant and only depends on the temperature and the pressure and whether it was formulated in the species-averaged biochemical expression (as in this work), and also on the pH. The progress of the enolase reaction was simulated by stepwise decreasing the substrate and increasing the product concentration. For each step, the activity coefficients of all reactants and products were predicted with ePC-SAFT and $K_{\gamma }$ was calculated. The resulting $K_{a}$ was compared with the known value and this procedure was repeated until the reaction equilibrium was reached (i.e., the known and calculated $K_{a}$ were equal). This yields the desired value of $K_{c}$, which was different for each set of reaction conditions (see Table S2 in supplementary materials). In order to calculate $K_{\gamma }$, two types of activity coefficients were used: the generic activity coefficient $\gamma_{i}$ and the rational activity coefficient $\gamma_{i}^{*}$. The standard state of $\gamma_{i}$ is the pure substance, while the standard state of $\gamma_{i}^{*}$ is the hypothetical ideal solution. In this work, we define the hypothetical ideal solution as a solution of 1 mol kg^−1^ of the substance diluted in water and an activity coefficient equal to that of the substance infinitely diluted in water (i.e., $\gamma_{i}^{*}=1$). $\gamma_{i}^{*}$ was used for the reacting agents because they are highly diluted in water. For this reason, the activity–coefficient ratio $K_{\gamma }$ was calculated according to Equation (6).

(6) $$K_{\gamma }=\frac{\gamma_{P}^{*,m,\mathrm{eq}}}{\gamma_{S}^{*,m,\mathrm{eq}}}$$

The hypothetical ideal solution is defined here as a solution of 1 mol kg^−1^ of the substance diluted in water. Furthermore, it was defined that the activity coefficients are equal to that of the substance infinitely diluted in water (i.e., $\gamma_{i}^{*}=1$).

The calculation of the entropy change ($\Delta_{R}S^{0,\;\mathrm{obs}}$ in J mol^−1^ K^−1^) is based on the Gibbs free energy change of reaction ($\Delta_{R}G^{0,\;obs}$ in J mol^−1^), the reaction enthalpy change ($\Delta_{R}H$ in J mol^−1^) and the measuring temperature T (in K), Equation (7).

(7) $$\Delta_{R}G^{0,\;\mathrm{obs}}=\;\Delta_{R}H-T\cdot \Delta_{R}S^{0,\;\mathrm{obs}}$$

The following equation applies at equilibrium, Equation (8):

(8) $$\Delta_{R}G^{0,\;\mathrm{obs}}=-R\;\cdot T\;\cdot {\mathrm{lnK}}_{c}$$

R, is the universal gas constant (J mol^−1^ K^−1^). Thus, the entropy change can be determined by using the equilibrium constants and the reaction enthalpy change determined in this work.

### 4.6. Kinetic Evaluation

Since we have studied the back reaction of F6P to G6P, i.e., in contrast to the direction of glycolysis, F6P is the substrate and G6P is the product of the reaction. The reaction rate as a function of the substrate concentration was determined from the thermal data according to the concept of Todd and Gomez [8]. This is briefly explained in the following. For the kinetic data, evaluation from ITC the substrate concentration, Equation (9) and the reaction rate $r_{S}\left(t\right)$, Equation (10) of each individual measuring point has to be calculated [8].

(9) cS(t)=cS0-∫0tP(t)·dt(ΔHR·m)

(10) rS(t)=P (t)ΔHR*m

Two models of irreversible thermodynamics, the Noor model and the flux-force model, were used for the kinetic data evaluation [31,32]. In part I, these models already provided an excellent description of the reaction progress of the reactions considered here and the temperature dependency of the kinetic constants fulfilled the Arrhenius relation [30]. From the general Noor Equation, three fit parameters are obtained. But it was shown that single saturation (either be the substrate or by the product) can reduce the number of parameters to two. For reaction 2, an enzyme saturation by the substrate was determined and the two parameters are $r_{max}$ and $K_{F6P}$ [30], Equation (11). The parameters can be obtained from a nonlinear fit of the plot r vs. $c_{S}^{}$. For reaction 9 an enzyme saturation by the product alone and not by the product and substrate was found to describe the data best. The parameters are ${\Lambda =c}_{E}\;\cdot \frac{k_{\mathrm{cat}}^{+}}{K_{\mathrm{PEP}}}$ and $K_{2PG}$ by excluding the double saturation, Equation (12) [30].

(11) $$\mathrm{For}\;\mathrm{reaction}\;2:\;r=r_{max}\cdot \frac{\frac{c_{F6P}}{K_{F6P}}}{1+\frac{c_{F6P}}{K_{F6P}}}\cdot (1-\frac{c_{F6P}\cdot \left(c_{F6P}^{0}{-c}_{F6P}^{\mathrm{eq}}\right)}{\left(c_{F6P}^{0}{-c}_{F6P}\right)\cdot c_{F6P}^{\mathrm{eq}}})$$

(12) $$\mathrm{For}\;\mathrm{reaction}\;9:\;r=\Lambda \cdot \frac{c_{\mathrm{PEP}}}{1+\frac{\left(c_{\mathrm{PEP}}^{0}{-c}_{\mathrm{PEP}}\right)}{K_{2\mathrm{PG}}}}\cdot (1-\frac{c_{\mathrm{PEP}}\cdot \left(c_{\mathrm{PEP}}^{0}{-c}_{\mathrm{PEP}}^{\mathrm{eq}}\right)}{\left(c_{\mathrm{PEP}}^{0}{-c}_{\mathrm{PEP}}\right)\cdot c_{\mathrm{PEP}}^{\mathrm{eq}}})$$

The flux-force model connects the reaction rates with the thermodynamic driving force ι by relating the total reaction rate r to the ratio of the sum of the reaction rates of the forward ($r^{+}$) and backward ($r^{-}$) reaction, Equation (13).

(13) $$\frac{r}{r^{+}{+r}^{-}}=\frac{r^{+}{-r}^{-}}{r^{+}{+r}^{-}}=\frac{1-\frac{r^{-}}{r^{+}}}{1+\frac{r^{-}}{r^{+}}}=\frac{{1-e}^{\Delta_{R}G/\mathrm{RT}}}{{1+e}^{\Delta_{R}G/\mathrm{RT}}}=\frac{\iota }{2-\iota }$$

$\Delta_{R}G$ is the Gibbs free energy change (in J mol^−1^). Neglecting the influence of the activity coefficients, the thermodynamic driving force ι is simplified as follows Equation (14) and (15):

(14) $$\mathrm{For}\;\mathrm{reaction}\;2:\;\iota =1-\;\frac{c_{G6P}\cdot c_{F6P}^{\mathrm{eq}}}{c_{F6P}\cdot {\;c}_{G6P}^{\mathrm{eq}}}$$

(15) $$\mathrm{For}\;\mathrm{reaction}\;9:\;\;\iota =1-\;\frac{c_{2\mathrm{PG}}\cdot c_{\mathrm{PEP}}^{\mathrm{eq}}}{c_{\mathrm{PEP}}\cdot {\;c}_{2\mathrm{PG}}^{\mathrm{eq}}}$$

Furthermore, the sum of the forward and backward reaction is correlated with the enzyme concentration and the phenomenological coefficient $L_{i}$ (s^−1^), since both reactions use the same active site, Equation (16).

(16) $${r=r}^{+}{+r}^{-}=L\cdot c_{E}$$

Combining equations 13 and 16 leads to the following final equation, Equation (17):

(17) $$r=L\cdot c_{E}\cdot \frac{\iota }{2-\iota }$$

The advantage of this approach is that the kinetic process is determined by only one adjustable parameter, which reflects the different cytosolic conditions. The phenomenological coefficient can be easily obtained from a linear regression of the plot with the rate against $\frac{\iota }{2-\iota }$ The slope corresponds to$\;L\cdot c_{E}$ and the enzyme concentration is known. Since this model only works close to equilibrium and the beginning of our calorimetric measurements is disturbed by the heat of dilution and the thermal inertia of the ITC, only the part $\frac{\iota }{2-\iota }<0.6$ was evaluated. An in-depth analysis of the influence of the ITC thermal inertia is given by Transtrum et al. [13].

## 5. Conclusions

Until now, glycolysis was often investigated under conditions that do not resemble those in the cell where it takes place. Furthermore, the reversibility of the reactions was often neglected. To improve this situation, we tested two models that take reversibility into account. Both Noor’s and the flux-force model show similar dependencies of the reaction rate on the cytosolic conditions. The models are therefore suitable for a wide range of cytosolic conditions. In addition, both models could be shown to work with an enzyme that has a uni-uni-mechanism and one with a uni-bi-mechanism. Unfortunately, Noor’s model provides neither significant parameters for several investigated conditions nor a clear dependence, which was ascribed to the different behaviors of the two parameters. To investigate the effect of cytosolic conditions, several cytosol constituents were added to the reaction and their effect was observed. Among the tested conditions, the concentrations of magnesium ions and crowding agents had the greatest influence, while temperature and pH-values had a medium influence on the reaction rate. Consequently, neglecting the cytosolic conditions leads to considerable errors when trying to describe the kinetics of processes in a cell. The influence of cytosolic conditions should also be considered when determining new standard data. Furthermore, the experiments carried out here only investigated individual reactions. An additional step that still has to be followed is the coupling of subsequent glycolytic reactions. With such measurements under cell conditions, a more realistic and comprehensive kinetic and thermodynamic re-examination of glycolysis should be conducted.

## Figures and Tables

**Table 1:** Comparison of measured and predicted reaction enthalpy change.

pH	$\Delta_{R}H\;(\mathrm{ITC})$	$\Delta_{R}H\;(\mathrm{Predicted})$
6	9.6 ± 0.2 kJ mol^−1^ (86 ± 2%)	13.2 kJ mol^−1^ (91%)
7	11.1 ± 0.5 kJ mol^−1^ (100%)	14.5 kJ mol^−1^ (100%)
8	10.8 ± 0.3 kJ mol^−1^ (97 ± 3%)	14.7 kJ mol^−1^ (101%)

**Table 2:** Entropy change values for both reactions with different crowding agents. Results for reaction 9 are from [6]. The indicated errors are from the triplicate measurements or the maximum error. The conditions not determined are marked with n.d.

	Gibbs Free Energy Change (kJ mol^−1^)		Reaction Enthalpy Change (kJ mol^−1^)		Entropy Change (J mol^−1^ K^−1^)	
Conditions	Reaction 2	Reaction 9	Reaction 2	Reaction 9 From [6]	Reaction 2	Reaction 9 From [6]
basic condition	2.8 ± 0.1	−13.7 ± 0.1	11.1 ± 0.5	2.4 ± 0.1	26.9 ± 1.7	51.8 ± 0.5
250 g L^−1^ PEG 20,000	12.8 ± 2.9	−10.8 ± 0.1	7.8 ± 0.3	0.7 ± 0.0	−15.9 ± 1.1	34.7 ± 0.8
250 g L^−1^ PEG 6000	12.8 ± 2.9	n.d.	7.7 ± 0.1	n.d.	−16.4 ± 0.4	n.d.
250 g L^−1^ BSA	6.4 ± 0.2	n.d.	7.4 ± 1.0	n.d.	3.1 ± 3.6	n.d.

**Table 3:** Cytosolic conditions varied by the experiments.

Conditions	Chemicals	Values	Unit
Temperature	-	298.15, 305.15, 310.15	K
pH	buffer	6, 7, 8	-
Na^+^ concentration	NaOH, NaCl	0.1, 0.15, 0.3	mol kg^−1^
Mg^2+^ concentration	MgCl_2_	1, 8, 15	mmol kg^−1^
Crowding agent concentration	PEG 20,000 PEG 6000 BSA	0, 113, 182, 250 250 250	g kg^−1^

## Supplementary Materials

Supplementary materials can be found at https://www.mdpi.com/1422-0067/21/21/7921/s1. References [60,61,62,63,64,65,66,67] are cited in the supplementary materials.

## Abbreviations

PEG	polyethylene glycol
BSA	bovine serum albumin
ITC	isothermal titration calorimetry
M-M	Michaelis-Menten
G6P	glucose-6-phosphate
F6P	fructose-6-phosphate
2PG	2-phosphoglycerate
PEP	phosphoenolpyruvate
PGI	phosphoglucose isomerase

## Symbols

**Symbol**	**Property**	**Unit**
r	reaction rate	mol kg^−1^ s^−1^
$c_{S}$	substrate concentration	mol kg^−1^
$c_{P}$	product concentration	mol kg^−1^
L	phenomenological coefficient/kinetic parameter	s^−1^
ι	thermodynamic driving force	-
$\Delta_{R}G$	Gibbs free energy change of biochemical reaction	J mol^−1^
$\Delta_{R}G^{o,\;\;\;obs}$	standard Gibbs free energy change of biochemical reaction	J mol^−1^
R	universal gas constant (8.314 J∙mol^−1^∙K^−1^)	J mol^−1^ K^−1^
T	temperature	K
$a_{i}$	activity of component i	-
$c_{i}$	concentration of component i	mol kg^−1^
$k_{cat}^{}$	kinetic constant of reaction	s^−1^
$K_{S/P}$	Michaelis constant for substrate/product	mol kg^−1^
$c_{i}^{0}$	concentration of component i at time 0	mol kg^−1^
$c_{i}^{eq}$	equilibrium concentration of component i	mol kg^−1^
$r_{max}$	maximum reaction rate	mol kg^−1^ s^−1^
Λ	kinetic parameter $\Lambda =\;\frac{r_{max}}{K_{S}}$	s^−1^
Q	heat	J
$\Delta_{R}H$	reaction enthalpy change of biochemical reaction	J mol^−1^
$\Delta_{R}S^{0,\;\;\;obs}$	standard entropy change of biochemical reaction	J mol^−1^ K^−1^
P	heat production rate	W
m	mass	kg
$K_{c}$	equilibrium-molality ratio of biochemical reaction	-/mol kg^−1^
$K_{a}$	thermodynamic equilibrium constant of biochemical reaction	-
$K_{\gamma }$	activity-coefficient ratio of biochemical reaction	-/mol kg^−1^
$\gamma_{i}^{*,m}$	rational activity coefficient of component i on molality base	-
$\varepsilon_{r}$	relative dielectric constant	-
