# Supplementary material for: Thermodynamics and Kinetics of Glycolytic Reactions. Part II: Influence of Cytosolic Conditions on Thermodynamic State Variables and Kinetic Parameters
Source: Int J Mol Sci. 2020 Oct 25;21(21):7921. doi: 10.3390/ijms21217921 (PMC7663428; doi:10.3390/ijms21217921)
Supplement: Supplementary file 1 [file ijms-21-07921-s001.pdf]

International Journal of Molecular Sciences

MDPI

Article

Supporting Material

Thermodynamics and Kinetics of Glycolytic Reactions. Part II: Influence of Cytosolic Conditions on Thermodynamic State Variables and Kinetic Parameters.

Kristina Vogel [1,3], Thorsten Greinert [2], Monique Reichard [1], Christoph Held [2,*], Hauke Harms [1] and Thomas Maskow [1,*]

[1] UFZ - Helmholtz Centre for Environmental Research, Dept. Environmental Microbiology, Leipzig, Permoserstr. 15, D-04318 Leipzig, Germany; thomas.maskow@ufz.de
[2] Laboratory of Thermodynamics, Department of Biochemical and Chemical Engineering, Technische Universitaet Dortmund, Emil-Figge-Str. 70, 44227 Dortmund, Germany; christoph.held@tu-dortmund.de
[3] Institute for Drug Development, Leipzig University Medical School, Leipzig University, Bruederstr. 34, 04103 Leipzig, Germany
* Correspondence: christoph.held@tu-dortmund.de (C.H.); thomas.maskow@ufz.de (T.M.)

Table of content

1. Chemicals used in this work 1
2. Parameter fits to the Noor Model 2
3. Influence of the sodium ion on the reaction rate 2
4. ePC-SAFT 3
5. Thermodynamic data of reaction 2 4
6. Thermodynamic data of reaction 9 [10] 6
7. Kinetic parameter of the Noor model at different cytosolic conditions 6
8. Kinetic parameter for the flux-force relationship at different cytosolic conditions 7
9. References .... **Error! Bookmark not defined.**

1. Chemicals Used in This Work

Table S1. Chemicals used in this work. Purities are given in percent by mass. Suppliers are: Sigma Aldrich (Sigma-Aldrich Chemie GmbH, Steinheim, Germany), Roth (Bernd Kraft, Duisburg, Germany), AppliChem (AppliChem GmbH, Darmstadt, Germany), CHEMSOLUTE (Th. Geyer GmbH & Co. KG,Renningen, Germany), Alfa Aesar (Thermo Fisher (Kandel) GmbH, Kandel, Germany).

Substance	CAS-number	M_i / g·mol^{-1}	Supplier	Purity [%]
Enolase (*Saccharomyces cerevisiae*)	9014-08-8	93,069	Sigma Aldrich	-
Phospho(enol)pyruvic acid	53823-68-0	190.02	Sigma Aldrich	98.9

monosodium salt hydrate				
Phosphoglucose isomerase type III from baker's yeast	9001-41-6	119,500	Sigma-Aldrich	-
Fructose 6 – phosphate disodium salt	26177-86-6	304.00	Alfa Aesar	95
MOPS	1132-61-2	209.27	AppliChem	≥ 99.5
Magnesium chloride hexahydrate	7791-18-6	203.3	AppliChem	-
Sodium chloride	7647-14-5	58. 4	CHEMSOLUTE	≥ 99
Sodium hydroxide	1310-73-2	40.00	Roth	≥ 98
Polyethylene glycol 20,000	25322-68-3	~20,000	Merck	-
Polyethylene glycol 6,000	25322-68-3	~6,000	Serva	-
Bovine serum albumin	9048-46-8	~66.000	Sigma Aldrich	-

2. Parameter Fits to the Noor Model

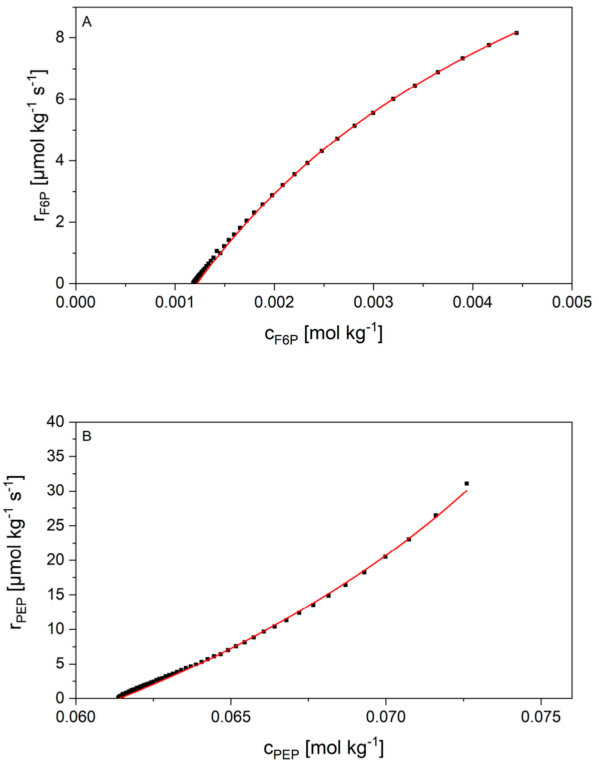

Figure S1. Fitting of the kinetic data (scatter) with the Noor model (red solid line) from [30]. A) shows the data of reaction 2 and B) the data of reaction 9. Both were measured under basic conditions.

3. Influence of the Sodium ion on the Reaction Rate

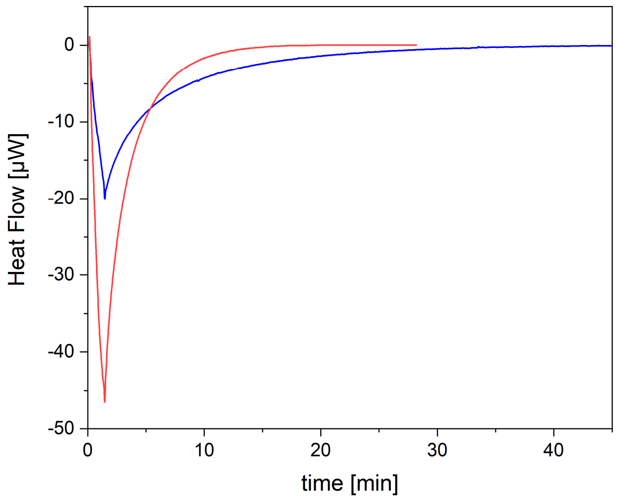

Figure S2. Influence of the sodium ions in the buffer on the reaction rate. Blue: measurements in the MOPS buffer with sodium ions, red: sodium ions were exchanged for potassium ions. The residual measurement conditions are those of the basic conditions. Sodium ions slow down the reaction.

4. ePC-SAFT

Activity coefficients were predicted with the equation of state ePC-SAFT. ePC-SAFT as proposed by Held et al. [60] is based on the original version of PC-SAFT from Gross and Sadowski [61]. It is a revised version of ePC-SAFT developed by Cameretti et al. [62] and allows accounting for interactions between anions and cations which are present in the reaction medium.

The prediction of activity coefficients or other thermodynamic properties with ePC-SAFT is based on the calculation of the residual Helmholtz energy A^{res} from different contributions that are shown in Equation S1.

$$A^{res}=A^{hc}+A^{disp}+A^{assoc}+A^{ion} \quad (S1)$$

The first of the four contributions is the Helmholtz energy A^{hc} of the hard-chain fluid, which is the reference fluid. The reference hard-chain system is itself composed of hard spheres. The other contributions account for perturbations to this reference fluid. The second contribution A^{disp} includes molecular dispersive interactions related to van der Waals forces. The third contribution A^{assoc} includes associative interactions related to hydrogen bonding forces. The fourth contribution A^{ion} includes ionic interactions and is described by a Debye-Hückel expression. The calculation of these four contributions with ePC-SAFT requires pure-component parameters and optionally binary parameters on top. Two of the pure-component parameters are the segment number m_i^{seg} and the segment diameter σ_i that describe the volume of the hard-chain reference system. Furthermore, the dispersion-energy parameter u_i/k_B including the Boltzmann constant k_B describes the dispersive interactions considered in A^{disp}. The hydrogen-bonding forces considered in A^{assoc} are described by the association-energy parameter $\varepsilon^{A_iB_i}/k_B$ and the association-volume parameter $\kappa^{A_iB_i}$. Last, the number of association sites N_i^{assoc} and the charge of an ion q are required.

Mixtures of substances are described using mixing rules to combine the pure-component parameters of the different substances. Lorentz-Berthelot [63] combining rules are used to determine the combined segment diameter σ_{ij} and the combined dispersion-energy parameter u_{ij}/k_B of components i and j according to Equation s. S2 and S3.

$$\sigma_{ij}=\frac{1}{2}(\sigma_i+\sigma_j) \quad (S2)$$

$$u_{ij}=\sqrt{u_i u_j}(1\text{-}k_{ij}) \quad (S3)$$

For mixtures, the binary interaction parameter k_{ij} is used to correct for deviations of u_{ij} from the geometric mean of u_i and u_j. Further, the association parameters $\varepsilon^{A_iB_j}$ and $\kappa^{A_iB_j}$ of two components i and j in a mixture were determined using the mixing rules proposed by Wolbach and Sandler [64].

$$\varepsilon^{A_iB_j}=\frac{1}{2}\left(\varepsilon^{A_iB_i}+\varepsilon^{A_jB_j}\right) \quad (S4)$$

$$\kappa^{A_iB_j} = \sqrt{\kappa^{A_iB_i}\kappa^{A_jB_j}}\left(\frac{\sqrt{\sigma_i\sigma_j}}{0.5(\sigma_i+\sigma_j)}\right)^3 \quad (S5)$$

Table S2. ePC-SAFT parameters applied in this work with the sources for the respective sets of parameters. For 2-PG the parameters of its isomer 3-PG were used.

	m_i^{seg}	σ_i	u_i/k_B	N_i^{assoc}	$\varepsilon^{A_iB_i}/k_B$	$\kappa^{A_iB_i}$	k_{i,H_2O}	z	source
	-	Å	K	-	K	-	-	-	
PEP	12.007	2.200	407.3	2+2	5000	0.1	a	-2	[64]
2-PG	3.110	4.660	322.0	5+5	501.2	10^{-4}	b	-2	[66]
G6P	22.329	2.227	243.3	5+5	5000	0.1	-0.095	-	[1], TW(kij)*
F6P	35.594	1.810	198.5	5+5	5000	0.1	-0.255	-	[1], TW(kij)*
MOPS	15.697	2.271	171.6	2+2	4418	0.001	-0.150	-	[64]
water	1.205	c	353.9	1+1	2426	0.0451	-	-	[67]
Na^{+} d	1	2.823	230.0	-	-	-	e	+1	[60]
Mg^{2+} f	1	3.133	1500	-	-	-	-0.25	+2	[60]
Cl^{-}	1	2.756	170.0	-	-	-	-0.25	-1	[60]

* TW(kij): the k_{ij} between $G6P^{2-}$ and $F6P^{2-}$ were refitted in this work to osmotic coefficient data from ref. [1] of water/$G6PK_2$ and water/$F6PK_2$ solutions, using the reference state infinite dilution in water.

a $k_{PEP,water}$ = -0.005083 T/K + 1.3316 [65]

b $k_{3-PG,water}$ = 0.002033 T/K - 0.7064 [66]

c σ_{water} = 2.7927 + 10.11 exp(-0.01775 T/K) - 1.417 exp(-0.01146 T/K) [67]

d k_{Na^+,Cl^-} = 0.3166 [60]

e $k_{Na^+,water}$= -0.007981 T/K + 2.3799 [60]

f k_{Mg^{2+},Cl^-} = 0.817 [60]

5. Thermodynamic Data of Reaction 2

Table S3. Values of the heat of reaction (Q), apparent biochemical equilibrium constant ($\boldsymbol{K_c}$) and calculated reaction enthalpy ($\Delta_R \boldsymbol{H}$) for each measured condition. Unless otherwise stated, the conditions were: T = 310.15 K, pH = 7, Na+ = 0.15 mol kg-1, Mg2+ = 1 mmol kg-1, PEG 20,000 = 0 mol kg-1

Influencing condition	Q [mJ]	K_c [mol kg^{-1}]	$\Delta_R H$ [kJ mol^{-1}]

temperature	298.15 K	7.63 ± 0.14	0.285	10.3 ± 0.2
	305.15 K	7.63 ± 0.07	0.318	10.6 ± 0.1
	310.15 K	7.86 ± 0.33	0.343	11.1 ± 0.5
	315.15 K	7.33 ± 0.11	0.369	10.5 ± 0.2
pH	6	7.13 ± 0.12	0.281	9.6 ± 0.2
	7	7.86 ± 0.33	0.343	11.1 ± 0.5
	8	7.50 ± 0.19	0.366	10.8 ± 0.3
sodium ion concentration	0.1 mol kg^{-1}	7.96 ± 0.05	0.362	11.4 ± 0.1
	0.15 mol kg^{-1}	7.86 ± 0.33	0.343	11.1 ± 0.5
	0.3 mol kg^{-1}	8.19 ± 0.06	0.298	11.2 ± 0.1
magnesium ion concentration	1 mmol kg^{-1}	7.86 ± 0.33	0.343	11.1 ± 0.5
	8 mmol kg^{-1}	7.76 ± 0.34	0.345	11.0 ± 0.4
	15 mmol kg^{-1}	7.56 ± 0.08	0.341	10.7 ± 0.1
PEG 20,000 concentration	0 g kg^{-1}	7.86 ± 0.33	0.343	11.1 ± 0.5
	113 g kg^{-1}	7.39 ± 1.02	0.054	8.2 ± 1.1
	182 g kg^{-1}	7.09 ± 0.22	0.019	7.6 ± 0.2
	250 g kg^{-1}	7.42 ± 0.26	0.007	7.8 ± 0.3
PEG 6,000 concentration	250 g kg^{-1}	7.27 ± 0.05	0.007	7.7 ± 0.1
BSA concentration	250 g kg^{-1}	6.42 ± 0.91	0.085	7.3 ± 1.0

6. Thermodynamic data of Reaction 9 [6]

Table S4. Values of the heat of reaction (*Q*), apparent biochemical equilibrium constant (K_c) and calculated reaction enthalpy ($\Delta_R H$) for each measured condition. Unless otherwise stated, the conditions were: T = 310.15 K, pH = 7, Na^+ = 0.15 mol kg^{-1}, Mg^{2+} = 1 mmol kg^{-1}, PEG 20,000 = 0 mmol kg^{-1}

Influencing condition		*Q* [mJ]	K_c [mol kg^{-1}]	$\Delta_R H$ [kJ mol^{-1}]
temperature	298.15 K	8.15 ± 0.72	239.4	2.4 ± 0.2
	305.15 K	7.85 ± 0.17	245.9	2.4 ± 0.1
	310.15 K	7.64 ± 0.22	251.3	2.4 ± 0.1
pH	6	11.74 ± 0.98	119.1	2.1 ± 0.2
	7	7.64 ± 0.22	251.3	2.4 ± 0.1
	8	13.37 ± 0.28	361.4	5.6 ± 0.1
sodium ion concentration	0.1 mol kg^{-1}	7.37 ± 0.05	249.9	2.3 ± 0.02
	0.15 mol kg^{-1}	7.64 ± 0.22	251.3	2.4 ± 0.1
	0.3 mol kg^{-1}	11.20 ± 1.02	251.1	3.5 ± 0.3
magnesium ion concentration	1 mmol kg^{-1}	7.64 ± 0.22	251.3	2.4 ± 0.1
	8 mmol kg^{-1}	7.74 ± 0.11	263.2	2.5 ± 0.04
	15 mmol kg^{-1}	8.19 ± 0.21	275.4	2.8 ± 0.1
PEG 20,000 concentration	0 g kg^{-1}	7.64 ± 0.22	251.3	2.4 ± 0.1
	113 g kg^{-1}	6.46 ± 0.19	155.8	1.4 ± 0.04
	182 g kg^{-1}	7.0 ± 0.93	121.6	1.3 ± 0.2
	250 g kg^{-1}	5.43 ± 0.11	77.5	0.7 ± 0.02

7. Kinetic Parameter of the Noor Model at Different Cytosolic Conditions

Table S5. Kinetic parameters of the Noor model for reaction 2 at different cytosolic conditions

Condition		r_{max} [µmol $kg^{-1}s^{-1}$]	K_{F6P} [mmol kg^{-1}]
temperature [K]	298.15 K	7.03 ± 0.58	4.18 ± 0.64
	305.15 K	10.30 ± 0.88	3.41 ± 0.39
	310.15 K	13.21 ± 1.20	3.70 ± 0.75
pH	6	5.66 ± 0.19	2.87 ± 0.19
	7	13.21 ± 1.20	3.70 ± 0.75
	8	19.81 ± 2.16	3.00 ± 0.62
sodium ion concentration	0.1 mol kg^{-1}	17.57 ± 0.32	3.17 ± 0.34
	0.15 mol kg^{-1}	13.21 ± 1.20	3.70 ± 0.75
	0.3 mol kg^{-1}	15.49 ± 0.57	3.36 ± 0.26
magnesium ion concentration	1 mmol kg^{-1}	13.21 ± 1.20	3.70 ± 0.75
	8 mmol kg^{-1}	10.46 ± 0.83	3.03 ± 0.24
	15 mmol kg^{-1}	19.92 ± 0.01	2.79 ± 0.27
PEG 20,000 concentration	0 g kg^{-1}	13.21 ± 1.20	3.70 ± 0.75
	113 g kg^{-1}	27.76 ± 1.10	6.22 ± 0.72
	182 g kg^{-1}	19.46 ± 0.23	5.26 ± 0.07
	250 g kg^{-1}	12.83 ± 0.12	7.73 ± 1.00
PEG 6,000 concentration	250 g kg^{-1}	27.11 ± 2.62	8.49 ± 0.63
BSA concentration	250 g kg^{-1}	17.48 ± 4.19	3.75 ± 0.96

Table S6. Kinetic parameters of the Noor model for reaction 9 at different cytosolic conditions

Condition		Λ [ms^{-1}]	K_{2PG} [mmol kg^{-1}]
temperature [K]	298.15 K	0.34 ± 0.01	10.2 ± 0.92
	305.15 K	0.44 ± 0.13	13.8 ± 3.35
	310.15 K	0.57 ± 0.03	16.5 ± 0.48
pH	6	0.31 ± 0.01	75.4 ± 39.5
	7	0.57 ± 0.03	16.5 ± 0.48
	8	0.72 ± 0.03	20.4 ± 2.78
sodium ion concentration	0.1 mol kg^{-1}	0.61 ± 0.07	14.1 ± 0.37
	0.15 mol kg^{-1}	0.57 ± 0.03	16.5 ± 0.48
	0.3 mol kg^{-1}	0.62 ± 0.06	17.0 ± 0.60
magnesium ion concentration	1 mmol kg^{-1}	0.57 ± 0.03	16.5 ± 0.48
	8 mmol kg^{-1}	0.91 ± 0.12	38.0 ± 4.88
	15 mmol kg^{-1}	1.10 ± 0.10	(-4.91 ± 5.69) 10^{16}
PEG 20,000 concentration	0 g kg^{-1}	0.57± 0.03	16.5 ± 0.48
	113 g kg^{-1}	0.49 ± 0.11	24.3 ± 5.44
	182 g kg^{-1}	0.29 ± 0.01	50.6 ± 39.20
	250 g kg^{-1}	0.11 ± 0.01	(1.56 ± 2.2) 10^{24}

8. Kinetic Parameter for the Flux-Force Relationship at Different Cytosolic Conditions

Table 7. The phenomenological parameter L of reaction 2 from the flux force relationship analysis at the different conditions.

Influencing condition.		*L* [s^{-1}]
temperature	298.15 K	217.18 ± 14.27
	305.15 K	358.80 ± 17.29
	310.15 K	518.44 ± 43.84
pH	6	224.93 ± 5.51
	7	518.44 ± 43.84
	8	834.74 ± 87.51
sodium ion concentration	0.1 mol kg^{-1}	720.26 ± 25.72
	0.15 mol kg^{-1}	518.44 ± 43.84
	0.3 mol kg^{-1}	572.06 ± 12.96
magnesium ion concentration	1 mmol kg^{-1}	518.44 ± 43.84
	8 mmol kg^{-1}	431.48 ± 47.17
	15 mmol kg^{-1}	845.79 ± 35.93
PEG 20,000 concentration	0 g kg^{-1}	518.44 ± 43.84
	113 g kg^{-1}	235.61 ± 19.11
	182 g kg^{-1}	85.45 ± 5.78
	250 g kg^{-1}	16.85 ± 2.70
PEG 6,000 concentration	250 g kg^{-1}	30.20 ± 1.75
BSA concentration	250 g kg^{-1}	327.63 ± 38.77

Table S8. The phenomenological coefficients *L* of reaction 9 from the flux force relationship at different cytosolic conditions.

Condition		*L* [s^{-1}]
temperature	298.15 K	10.13 ± 0.65
	305.15 K	14.57 ± 3.20
	310.15 K	20.37 ± 1.03
pH	6	11.59 ± 0.44

	7	20.37 ± 1.03
	8	29.60 ± 1.78
sodium ion concentration	0.1 mol kg^{-1}	21.17 ± 2.51
	0.15 mol kg^{-1}	20.37 ± 1.03
	0.3 mol kg^{-1}	22.22 ± 2.10
magnesium ion concentration	1 mmol kg^{-1}	20.37 ± 1.03
	8 mmol kg^{-1}	38.24 ± 4.66
	15 mmol kg^{-1}	56.10 ± 5.79
	0 g kg^{-1}	20.37 ± 1.03
PEG 20,000 concentration	113 g kg^{-1}	15.70 ± 2.69
	182 g kg^{-1}	8.98 ± 1.38
	250 g kg^{-1}	2.69 ± 0.23

© 2020 by the authors. Licensee MDPI, Basel, Switzerland. This article is an open access article distributed under the terms and conditions of the Creative Commons Attribution (CC BY) license (http://creativecommons.org/licenses/by/4.0/).
